# Grafts

**Published:** 1891-01

**Authors:** Frank S. Davis

**Affiliations:** Quincy, Mass.


### GRAFTS.

Editors Homœopathic Physician:

I have read with interest the articles in the *Homœopathic Recorder*, "How Hahnemann Cured," and "Grafts," also recently in Homœopathic Physician, November, 1890, article entitled, "Grafts," by Dr. W. A. Yingling.

I desire to add my word of experience in confirmation of Dr. Yingling's experience. I have used, during the last ten years of active practice, the 200 (B. & T.) almost entirely and with success.

For the last two years have been using grafts (Dr. Swan's), CM, CMM, DM, DMM. I never have had any trouble in "labor cases," have never used instruments to deliver. I use only the indicated remedy. I have never had a fatal case in confinement or a ruptured perineum.

Have never lost a case of typhoid fever or pneumonia.

My success has been more marked since using the higher potencies. I usually give a few powders of the indicated remedy and follow with Sac-lac. I try to make sure I have the indicated remedy and then permit the improvement to continue without change of remedy or a second dose. If every one who doubts would make the practical test of our law of cure as Hahnemann taught it they would find success greater with "grafts" than with the low dilutions. Faithful tests will give proof of the truth and help to hold high the banner of *strict Homœopathy.*

Close study to individualize each case, getting the characteristic, peculiar conditions concomitant with the general symptoms of cases, and selecting carefully from those remedies which cover those peculiarities, is the only way I know of to cure any sickness with certainty. Such study will give confidence to wait for a cure which will satisfy the doubtful mind as to whether or not *grafts cure.*

Experience will bring evidence if trial is made honestly and after careful study. Let us have more upon this subject of "grafts." I hope to hear from some having more experience.

Very truly,

QUINCY, MASS., November 7th. FRANK S. DAVIS.

